# Supplementary material for: Oncogene expression from extrachromosomal DNA is driven by copy number amplification and does not require spatial clustering in glioblastoma stem cells
Source: eLife. 2022 Dec 7;11:e80207. doi: 10.7554/eLife.80207 (PMC9728993; doi:10.7554/eLife.80207)
Supplement: Figure 1—source data 1. — Median values for number of EGFR DNA FISH signals per metaphase spread for E26 and E28 cell lines. Data are for Figure 1D. Mean Chr7 (Texas Red) and EGFR (FITC) DNA FISH signal intensity in bins eroded from the periphery (1) to the centre (5) of the nucleus of neural stem cell (NSC), E26 and E28 cells. p-Values from Kruskall-Wallis test. Data are for Figure 1G and Figure 1—figure supplement 1. [file elife-80207-fig1-data1.docx]

**Figure 1 – Source Data 1**

Source data for Figure 1D

| **Cell line (n)** | Median | Mann-Whitney test |
| --- | --- | --- |
| E26 (25) | 51 | p = 0.008 |
| E28 (24) | 12 |  |

Source data for Figure 1G and Figure Supplement 1

|  |  | Bin | | | | | Kruskall-  Wallis |
| --- | --- | --- | --- | --- | --- | --- | --- |
| **Cell line** (n) | Signal | 1 | 2 | 3 | 4 | 5 |  |
| **NSC** (66) | chr 7 (TxR) | 0.6159 | 0.5656 | 0.5771 | 0.5399 | 0.4732 | p <0.0001 |
| **E26** (59) |  | 0.4292 | 0.3699 | 0.3279 | 0.2979 | 0.2665 | p <0.0001 |
| **E28** (64) |  | 0.6563 | 0.6023 | 0.5655 | 0.4960 | 0.4175 | p <0.0001 |
| **NSC** | EGFR (FITC) | 0.3301 | 0.2475 | 0.2131 | 0.1991 | 0.1972 | p <0.0001 |
| **E26** |  | 0.2625 | 0.2034 | 0.1838 | 0.1802 | 0.1661 | p <0.0001 |
| **E28** |  | 0.3235 | 0.2554 | 0.2236 | 0.2117 | 0.2048 | p <0.0001 |
| **NSC** | FITC:TxR | 0.5541 | 0.4389 | 0.3918 | 0.3756 | 0.3867 | p <0.0001 |
| **E26** |  | 0.6095 | 0.5573 | 0.5715 | 0.6239 | 0.6618 | p = 0.0598 |
| **E28** |  | 0.4802 | 0.4208 | 0.4105 | 0.4207 | 0.4682 | p = 0.0117 |
