# Supplementary material for: Oncogene expression from extrachromosomal DNA is driven by copy number amplification and does not require spatial clustering in glioblastoma stem cells
Source: eLife. 2022 Dec 7;11:e80207. doi: 10.7554/eLife.80207 (PMC9728993; doi:10.7554/eLife.80207)
Supplement: Figure 2—source data 1. — Statistical analysis of data for Figure 2—figure supplement 1B-E. Mean shortest interprobe distance and shortest interprobe distance in E26 and E28 cell lines. EGFR-EGFR interprobe distance (μm) = median values shown. The statistical significance of the data distributions between E26 and E28 were assessed with a Mann-Whitney test. n = number of nuclei. [file elife-80207-fig2-data1.docx]

#### **Figure 2 – Source Data 1**

Source data for Figure Supplement 1

|  | Cell line | | Mann-Whitney test |
| --- | --- | --- | --- |
|  | **E26** (n) | **E28** (n) |  |
| **Mean shortest interprobe distance** (μm) | 1.513 (37) | 2.092 (36) | p = 0.1326 |
| **Mean shortest interprobe distance** (μm where number of foci >10 per nucleus | 0.8958 (18) | 1.136 (9) | p = 0.0052 |
| **Shortest interprobe distance** (μm) | 0.470 (37) | 1.315 (36) | p = 0.0322 |
| **Shortest interprobe distance** (μm) where number of foci >10 per nucleus | 0.3156 (18) | 0.4145 (9) | p = 0.1450 |
