# Supplementary material for: Oncogene expression from extrachromosomal DNA is driven by copy number amplification and does not require spatial clustering in glioblastoma stem cells
Source: eLife. 2022 Dec 7;11:e80207. doi: 10.7554/eLife.80207 (PMC9728993; doi:10.7554/eLife.80207)
Supplement: Figure 3—source data 1. — Median number of CDK4 and PDGFRA DNA FISH foci in E25 cell line (n=26) nuclei. Data are for Figure 3—figure supplement 1B. Mean shortest interprobe distance and shortest interprobe distance between CDK4 and PDGFRA DNA FISH foci in E25 cell line. Statistical analysis of data for Figure 3—figure supplement 1C and D, Interprobe distance (μm) between fosmids indicated = median values shown. Value in brackets indicates adjusted p-value (adj) = Bonferroni. n=26 nuclei. [file elife-80207-fig3-data1.docx]

**Figure 3 – Source Data 1**

Source data for Figure Supplement 1B

| **E25 (n=26)** | Median number of foci |
| --- | --- |
| CDK4 | 33 |
| PDGFRA | 17.5 |

Source data for Figure Supplement 1C and 1D

|  |  | | | | Kruskall-Wallis | Mann-Whitney test  CDK4-CDK4 vs CDK4-PDGFRA |
| --- | --- | --- | --- | --- | --- | --- |
| E25 (n=26) | **CDK4-**  **CDK4** | **PDGFRA-PDGFRA** | **CDK4-**  **PDGFRA** | **PDGFRA-CDK4** |  |  |
| **Mean shortest interprobe distance** (μm) | 1.270 | 2.108 | 2.292 | 1.713 |  |  |
| **Shortest interprobe distance** (μm) | 0.3423 | 0.5650 | 0.3380 |  | p = 0.0001 | p= 0.9494 (1.00) |
