# Supplementary material for: Oncogene expression from extrachromosomal DNA is driven by copy number amplification and does not require spatial clustering in glioblastoma stem cells
Source: eLife. 2022 Dec 7;11:e80207. doi: 10.7554/eLife.80207 (PMC9728993; doi:10.7554/eLife.80207)
Supplement: Figure 4—source data 1. — EcDNA-large RPB1 foci distances for neural stem cell (NSC), E26 and E28 cell lines. Statistical analysis of data for DNA-ImmunoFISH (i – Figure 4E and F), RNA-ImmunoFISH (ii – Figure 4—figure supplement 1E F) EcDNA-large RPB1 foci distance (μm) indicated = median values shown. (iii) Median number of EGFR RNA FISH signals for NSC, E26 and E28 cell lines. (iv) Median ecDNA-large POLR2G foci distances for E28 mCherry-POLR2G cell line (Figure 4—figure supplement 1I, J). n = number of nuclei. Kruskall-Wallis and Mann-Whitney tests performed with comparisons as indicated. [file elife-80207-fig4-data1.docx]

**Figure 4 – Source Data 1**

Source Data for Figure 4 and Figure Supplement 1 as annotated

|  | Cell line | | | Kruskall-Wallis | Mann-Whitney test |
| --- | --- | --- | --- | --- | --- |
|  | **NSC** (n) | **E26** (n) | **E28** (n) |  |  |
| 1. **Immuno-FISH (DNA) (Figure 4E and 4F)** | | | | | |
| Mean shortest ecDNA-condensate distance (EGFR DNA-Rpb1) (μm) | 3.950 (7) | 2.995 (8) | 1.970 (7) | p=0.1269 |  |
| Shortest ecDNA-condensate distance (EGFR DNA-Rpb1) (μm) | 2.920 (7) | 2.600 (8) | 1.900 (7) | p=0.5234 |  |
| 1. **Immuno-FISH (RNA) (Figure 4 Figure Supplement 1E and 1F)** | | | | | |
| Mean shortest ecDNA-condensate distance (EGFR RNA-Rpb1) (μm) |  | 4.667 (10) | 5.008 (12) |  | p=0.6277 |
| Shortest ecDNA-condensate distance (EGFR RNA-Rpb1) (μm) |  | 1.766 (10) | 2.437 (12) |  | p=0.1802 |

1. Source data for Figure Supplement 1A

| **Cell line (n)** | Median number of EGFR RNA FISH signals | Mann-Whitney (vs NSC) | Mann-Whitney (vs E28) |
| --- | --- | --- | --- |
| NSC (25) | 1 |  |  |
| E26 (29) | 5 | <0.0001 | 0.001 (0.003) |
| E28 (30 | 2 | 0.0174 (0.052) |  |

1. Source data for Figure Supplement 1H and 1I

| **Immuno-FISH** | **E28 mCherry-PolR2G (n)** |
| --- | --- |
| Mean shortest ecDNA-condensate distance (μm) | 4.804 (14) |
| Shortest ecDNA-condensate distance (μm) | 1.561 (14) |
