# Supplementary material for: Oncogene expression from extrachromosomal DNA is driven by copy number amplification and does not require spatial clustering in glioblastoma stem cells
Source: eLife. 2022 Dec 7;11:e80207. doi: 10.7554/eLife.80207 (PMC9728993; doi:10.7554/eLife.80207)
Supplement: Figure 5—source data 1. — (i) RNA:DNA FISH EGFR foci ratios. Statistical analysis of data for Figure 5B, RNA:DNA FISH EGFR foci ratio = mean values shown. n = number of nuclei, total across three biological replicates. Values in brackets indicate adjusted p-value (adj) = Bonferroni. (ii) Correlation of RNA:DNA ratio and ecDNA/total foci ratio (Figure 5C), Spearman r (p-value) shown for three biological replicates. N = number of nuclei. Rep1 data shown in figure. (iii) RNA-seq/whole genome sequencing (WGS) allele frequency ratio, for Figure 5E. Median and number of SNPs per gene per cell line. (iv) EcDNA versus chromosomal EGFR exons (Figure 5F and G), Mann-Whitney test of normalized RNA counts between chromosomal and predominantly EGFR ecDNA exons. (v) Mann-Whitney test of of EGFR RNA FISH foci in FACs sorted E26 and E28 cells (Figure 5—figure supplement 1E). [file elife-80207-fig5-data1.docx]

**Figure 5 – Source Data 1**

1. Source data for Figure 5B

| Cell line (n) | **RNA: DNA FISH ratio (mean)** | **One-way ANOVA** | **Unpaired T-test (vs E26)** | **Unpaired T-test (vs E28)** |
| --- | --- | --- | --- | --- |
| **NSC** (67) | 0.3419 | p=0.0189 | p=0.005 (0.015) | p = 0.1118 (0.3354) |
| **E26** (98) | 0.6037 |  |  |  |
| **E28** (95) | 0.4960 |  | p = 0.1885 (0.5655) |  |

1. Source data for Figure 5C

|  | **Spearman r (p value)** | | |
| --- | --- | --- | --- |
| Cell line (n) | **Rep1** | **Rep2** | **Rep3** |
| **E26 (92)** | -0.005807 (0.9761) | 0.1878 (0.4150) | 0.01168 (0.9415) |
| **E28 (93)** | -0.02923 (0.8598) | -0.07586 (0.7372) | 0.2892 (0.1083) |

1. Source data for Figure 5E

|  | **Median (Number of SNPs per gene)** | | |
| --- | --- | --- | --- |
| Cell line | **VSTMA** | **EGFR** | **LANCL2** |
| **E26** |  | 0.9789 (3) | 0.9272 (2) |
| **E28** | 1.0484 (1) | 1.003 (8) | 0.7776 (1) |

1. Source data for Figure 5F and 5G

| Cell line | **Mann-Whitney Test (ecDNA vs chrom exons)** |
| --- | --- |
| **E26** | p = 0.3090 |
| **GBM39** | p = 0.0517 |

1. Source Data for Figure Supplement 1E

|  | **Median number of EGFR foci (number of nuclei)** | | **Mann-Whitney test** |
| --- | --- | --- | --- |
| Cell line | EGFR High | EGFR Low |  |
| E26 | 58 (35) | 8 (30) | <0.0001 |
| E28 | 11 (29) | 3 (30) | <0.0001 |
