# Supplementary material for: Oncogene expression from extrachromosomal DNA is driven by copy number amplification and does not require spatial clustering in glioblastoma stem cells
Source: eLife. 2022 Dec 7;11:e80207. doi: 10.7554/eLife.80207 (PMC9728993; doi:10.7554/eLife.80207)
Supplement: Supplementary file 1. — (A)Fosmid probes for DNA FISH related to STAR methods. Genome coordinates (Mb) are from the hg38 assembly of the human genome. (B) CrRNA sequence and dsDNA sequence for mCherry_PolR2G CRISPR knock-in. [file elife-80207-supp1.docx]

**Methods – Supplementary File 1**

#### A) Fosmid probes for DNA FISH

| Locus | Gene | Start (bp) | End (bp) | Fosmid ID | Clone name |
| --- | --- | --- | --- | --- | --- |
| 7: 55019017-55211628 | EGFR | 55024189 | 55063180 | G248P88704G2 | WI2-2910M03 |
| 12: 57747727-57752310 | CDK4 | 57746054 | 57783419 | G248P80931E4 | WI2-0793J08 |
| 4: 54,229,293-54298245 | PDGFRA | 54230802 | 54268615 | G248P86466H11 | WI2-2022O22 |

#### B) CrRNA sequence and dsDNA sequence for mCherry_PolR2G CRISPR knock-in

| Locus | crRNA | dsDNA donor DNA block |
| --- | --- | --- |
| POLR2G | ACCAAGGGTAGGAGGCCACC | \| TCCCTGATGGACGATTACTTGGGTGAGTGCCTGATCATAGGTGCTGGGGTTATTGCCTGGAGAAGGGATGTGTGGGGGTGGGGAGTAATATAGGATTCAATGCCCAAATCAGAGAGACAGAAGAAACTTTCATGCTGTCTGCTTGAAAGATCCAGGACATTTGCCTTGGGATGAGGAGTACATGGTTGTGGCTACCCTAAATTCCGGTTCTAACTGATATGCTTTTTCTGGTTTCGCAGGGCTTGTAAGCGGCGACGGCGGCAGCGGCGGCGGCAGCATGGTGAGCAAGGGCGAGGAGGATAACATGGCCATCATCAAGGAGTTCATGCGCTTCAAGGTGCACATGGAGGGCTCCGTGAACGGCCACGAGTTCGAGATCGAGGGCGAGGGCGAGGGCCGCCCCTACGAGGGCACCCAGACCGCCAAGCTGAAGGTGACCAAGGGTGGCCCCCTGCCCTTCGCCTGGGACATCCTGTCCCCTCAGTTCATGTACGGCTCCAAGGCCTACGTGAAGCACCCCGCCGACATCCCCGACTACTTGAAGCTGTCCTTCCCCGAGGGCTTCAAGTGGGAGCGCGTGATGAACTTCGAGGACGGCGGCGTGGTGACCGTGACCCAGGACTCCTCCCTGCAGGACGGCGAGTTCATCTACAAGGTGAAGCTGCGCGGCACCAACTTCCCCTCCGACGGCCCCGTAATGCAGAAGAAGACCATGGGCTGGGAGGCCTCCTCCGAGCGGATGTACCCCGAGGACGGCGCCCTGAAGGGCGAGATCAAGCAGAGGCTGAAGCTGAAGGACGGCGGCCACTACGACGCTGAGGTCAAGACCACCTACAAGGCCAAGAAGCCCGTGCAGCTGCCCGGCGCCTACAACGTCAACATCAAGTTGGACATCACCTCCCACAACGAGGACTACACCATCGTGGAACAGTACGAACGCGCCGAGGGCCGCCACTCCACCGGCGGCATGGACGAGCTGTACAAGTGAGGCTGGTGCCCTCCTACCCTTGGTCCTACTCTAGGAAGTGTGATTGTCACACTTATCATGTTGTCCAGAGGTCCAGTCTGGCTGCTGTTGTGGAGGCAAGGAAGGCAACTCATCCCAGAAGGCATCTGGTGCTTCTTGTAGCTTAACTACTGCCTCCTCATTTTTCAGTATGTGTTCTAAGTATAAAAAGTCCTTGG \| \| --- \| |

A) Fosmid probes for DNA FISH Related to STAR Methods. Genome co-ordinates (Mb) are from the hg38 assembly of the human genome. B) CrRNA sequence and dsDNA sequence for mCherry_PolR2G CRISPR knock-in.
